# Interosseous impasse: a unique case of bullet lodgement between the radius and ulna

**DOI:** 10.1093/jscr/rjaf036

**Published:** 2025-02-05

**Authors:** Rand Y Omari, Shiyas Mohammedali, Sohail J Quazi, Mohammed El-Debs, Ahmad Y Al-Omari, Mazin Mohammed, Mohammed Muneer

**Affiliations:** Plastic and Reconstructive Surgery Department, Hamad Medical Corporation, Ahmed Bin Ali Street, Doha, Qatar; Plastic and Reconstructive Surgery Department, Hamad Medical Corporation, Ahmed Bin Ali Street, Doha, Qatar; Plastic and Reconstructive Surgery Department, Hamad Medical Corporation, Ahmed Bin Ali Street, Doha, Qatar; Plastic and Reconstructive Surgery Department, Hamad Medical Corporation, Ahmed Bin Ali Street, Doha, Qatar; Plastic and Reconstructive Surgery Department, Hamad Medical Corporation, Ahmed Bin Ali Street, Doha, Qatar; Plastic and Reconstructive Surgery Department, Hamad Medical Corporation, Ahmed Bin Ali Street, Doha, Qatar; Plastic and Reconstructive Surgery Department, Hamad Medical Corporation, Ahmed Bin Ali Street, Doha, Qatar

**Keywords:** upper limb injuries, gunshot injuries, bullet entrapment, reconstructive surgery, trauma surgery, ballistic injuries, bullet extraction, hand surgery

## Abstract

Gunshot injuries to the upper limb are complex, particularly when a projectile lodges between the radius and ulna. These cases demand a detailed approach due to the complicated anatomy and potential for severe functional impairment. A 42-year-old female presented with severe right upper limb pain and motor impairment following a gunshot injury. The bullet entered through the posterior shoulder, shattered the mid-humerus and proximal radius, and lodged between the distal radius and ulna. Multidisciplinary surgical intervention included external fixator application and removal, open reduction, and internal fixation of the humerus, complete nerve decompression, and vascular repair using an interposition reversed saphenous vein graft. The patient’s postoperative course was uneventful, and follow-up showed sensory improvement and some motor recovery. This case highlights the complexity of managing gunshot injuries with bullets lodged between the radius and ulna. Multidisciplinary care is essential for addressing the varied challenges posed by such injuries. Further research is needed to refine treatment strategies and improve patient outcomes in similar cases.

## Introduction

Ballistic injuries involving the forearm are a common occurrence in trauma care; however, bullet retention between the ulna and radius is an uncommon and underexplored phenomenon. While gunshot wounds to the extremities are widely covered in the literature, specific attention to projectiles lodged in the interosseous region remains limited [1, 2]. In this article, we describe a rare case of a bullet retained between the ulna and radius following an atypical trajectory. By highlighting the clinical presentation, surgical decision-making, and postoperative considerations, this report aims to provide a comprehensive framework for managing similar ballistic injuries while addressing the broader implications of retained bullets in the upper limb.

## Case report

A 42-year-old female presented to our hospital with severe diffuse right upper limb pain, hyperesthesia, and diffuse motor impairment after she suffered a gunshot injury to her right arm during a recent conflict. She underwent external fixation to stabilize the humerus fracture in another country and subsequently presented to our emergency department 2 weeks after injury. She has no co-morbidities and was not taking any medications regularly. Physical examination revealed severely impaired flexion and extension of the wrist and all fingers, diminished range of motion of the elbow and shoulder, indicating the probable extent of the nerve injury. The entry point of the projectile was identified at the posterior shoulder, with no corresponding exit point. Additionally, varying degree of motor and sensory impairment was noted in all three peripheral nerves distribution, notably in radial nerve, accompanied by superficial burns over the dorsal hands.

**Figure 1:**
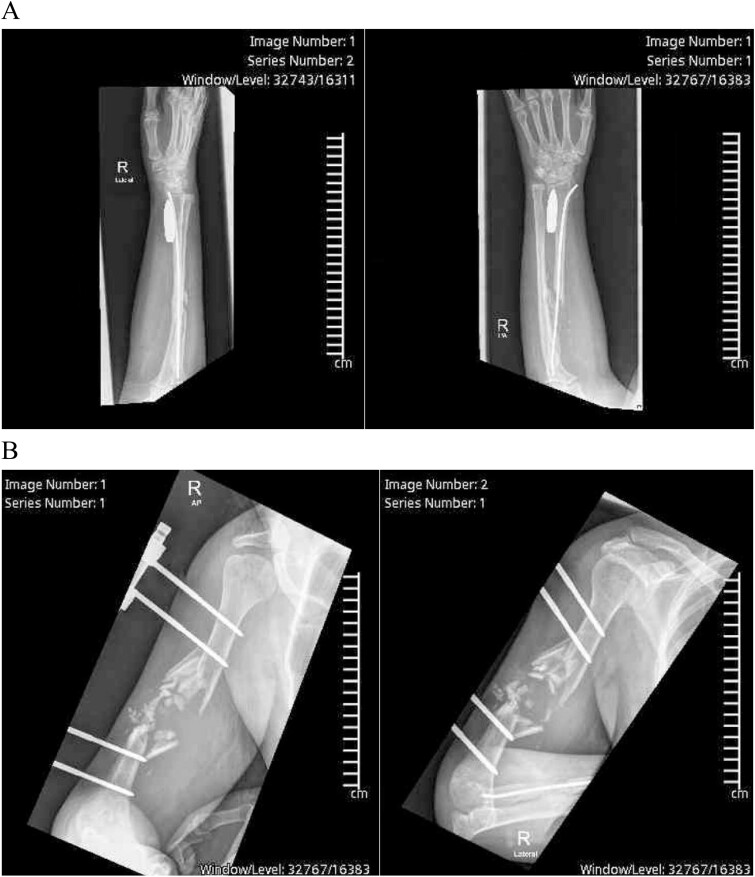
(A) Preoperative X-ray showing the bullet lodgment along the volar aspect of the distal radius. (B) Preoperative X-ray showing the external fixation device with pins at the proximal and distal ends of the right humerus with shattered mid-third of the shaft, surrounded by displaced fracture fragments, which was shattered along the bullet’s tract before settling in the wrist.

Right upper limb X-rays revealed external fixation device with pins at the proximal and distal ends of the right humerus with shattered mid-third of the shaft, surrounded by displaced fracture fragments, crushed proximal radius fracture with intramedullary nailing noted in satisfactory position, and a 5 × 1 cm bullet was seen lying longitudinally along the volar aspect of the distal radius (Fig. 1A and B). The carpal bones alignment was maintained A plain CT scan of the upper limb showed the comminuted fracture of the right humerus with multiple bone fragments and displacement.

The patient was managed by a multidisciplinary team comprising plastic, orthopedic, and vascular surgery teams. She underwent external fixator removal with open reduction and internal fixation of the right humerus fracture by Orthopedic Surgery.

**Figure 2:**
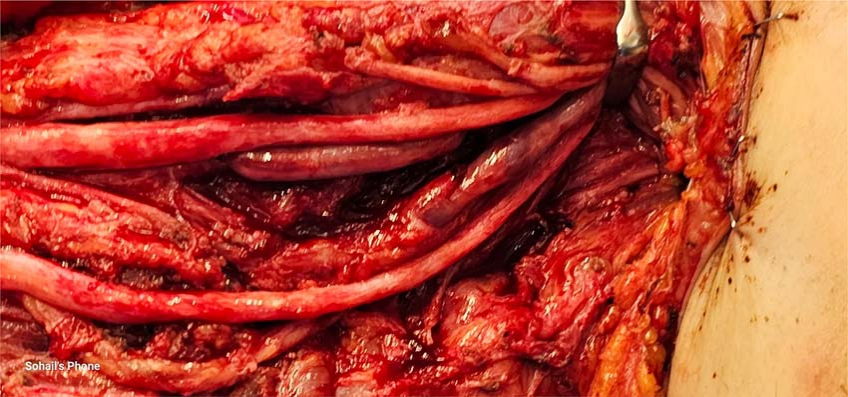
Intraoperative X-ray showing severe adhesions of the radial, median and ulnar nerves in the proximal 2/3rd of the arm, adhesions were lesser as the nerves coursed distally in the lower arm and proximal forearm.

**Figure 3:**
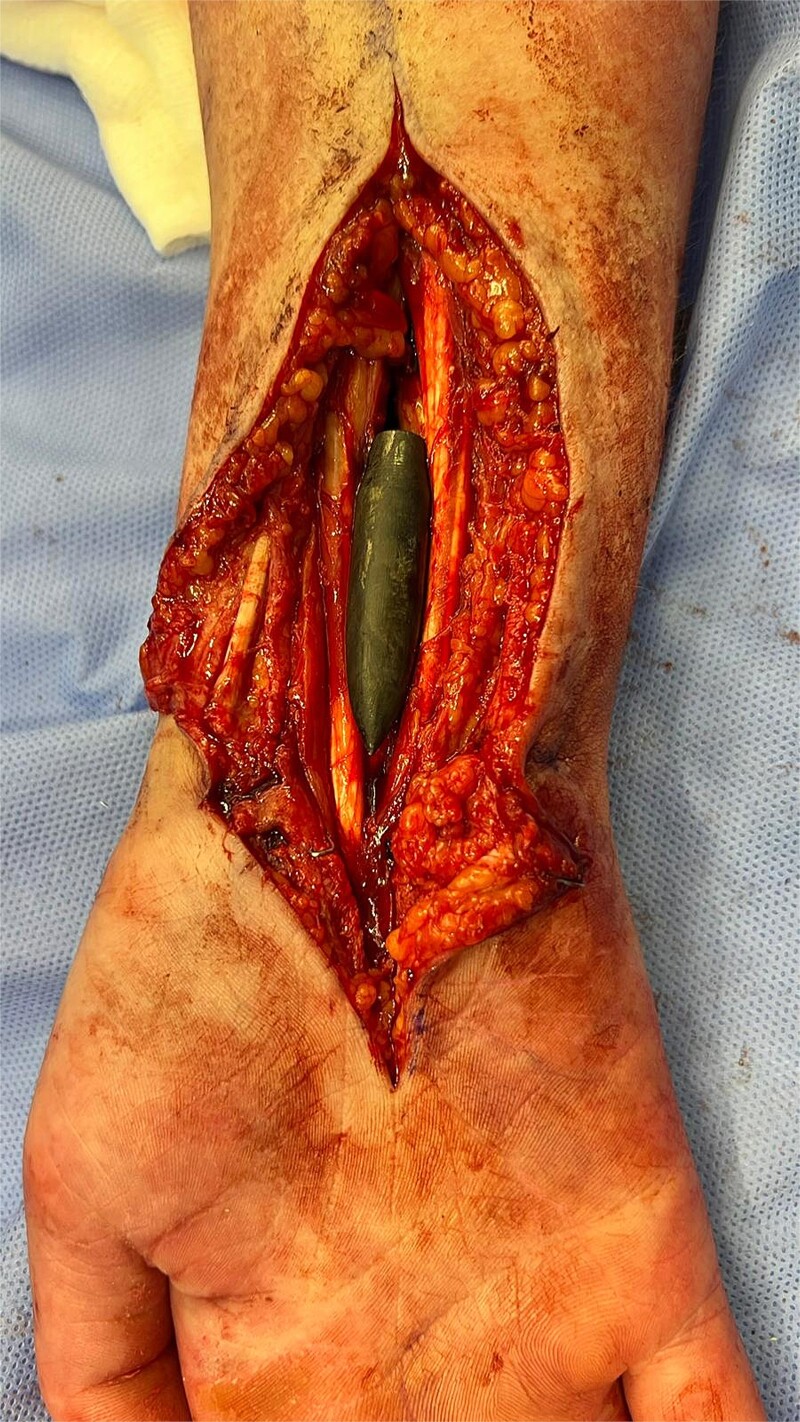
Intraoperative X-ray showing the final position of the bullet on the volar aspect of the radius.

**Figure 4:**
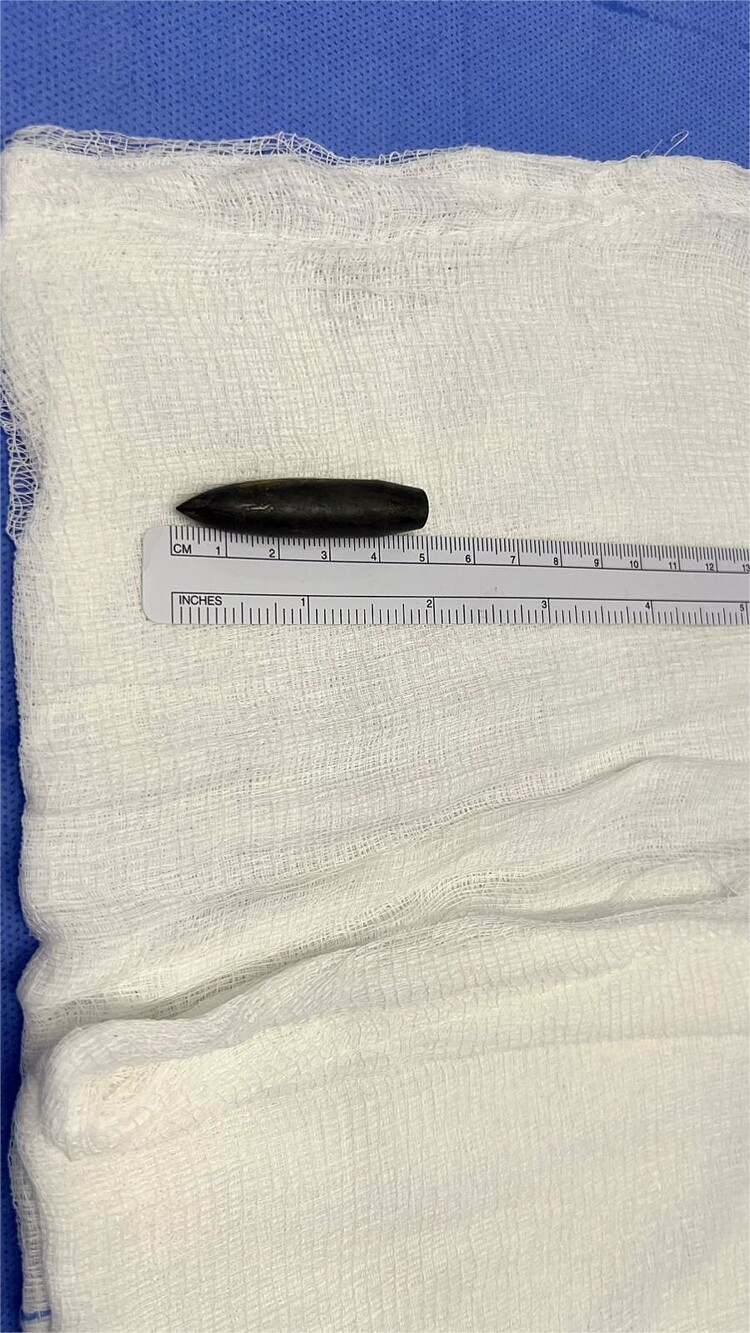
The bullet post extraction with a length of 5 centimeters.

Exploration revealed severe adhesions of radial, median, and ulnar nerves in the proximal 2/3rd third of the arm, adhesions were lesser as the nerves coursed distally in the lower arm and proximal forearm. The radial nerve was completely severed with a marked gap between the distal and proximal ends (Fig. 2). Muscle bellies lying along the bullet tract, at the posterior compartment of arm and volar forearm were charred, fibrotic and replaced by fat. Complete decompression of the peripheral nerves in addition to bullet extraction was performed by Plastic Surgery, with a plan for tendon transfer in regard to the severed radial nerve later on. (Figs 3 and 4) During the surgery, the brachial artery was found severely attenuated, matted to the venae comitantes, and stuck to the bone at fracture site without any recognizable plane in-between, and during adhesiolysis, the artery was inadvertently injured. It was repaired using interposition reversed saphenous vein graft by Vascular Surgery. The patient’s postoperative period was uneventful, with good distal capillary refill and pain control. She was discharged 8 days after the surgery and regularly followed in the outpatient clinic and the hand occupational therapy unit. 3 months follow-up showed significant improvement in sensory symptoms and minimal improvement in motor function.

## Discussion

Bullet injuries lodged between the ulna and radius represent a significant yet underexplored aspect of ballistic trauma [1]. While the literature extensively covers gunshot wounds to various anatomical regions, including extremities, the specific incidence and clinical implications of bullets lodged between these two forearm bones remain scarce [2]. The case presented here is a unique case of a retained bullet in the upper limb with an atypical trajectory, it provides an example of the management for a non-fatal gunshot wound to the upper limb to be used as a template for future care with a similar case.

The prevalence of bullet injuries in the upper limb likely varies depending on multiple factors, including the mechanism of injury, ballistic characteristics of the projectile, and environmental circumstances [3]. In close-range shootings where bullets may fragment or deform upon impact, the likelihood of lodging between the ulna and radius may increase. Conversely, in cases of high-velocity projectiles or shots fired from a distance, the trajectory may be less likely to result in such entrapment [4]. What is unique about the case presented here is that the projectile was shot from a long distance, while the patient was sitting at home, it entered through the posterior shoulder, shattered the mid humerus and proximal radius, seared all the muscles along its trajectory, to finally settle in its location at distal radio-ulnar joint.

Surgeons often face complex decisions regarding whether bullet extraction is warranted or if a more conservative approach may be preferable to minimize further harm. The decision to extract or leave bullets in a patient depends on factors such as the bullet’s location, potential risks of removal, and the patient’s condition. Upper extremity retained bullets often lead to complex injuries that involve nerve, bone, tendon, and vessel injury due to the tight spaces in this location [5]. In general, retained bullets causing restricting motion, causing nerve impingement, located within a vessel, or located subcutaneously within the hand or wrist, should all be removed [6].

The long-term implication of retained bullets in the upper limb remains an area of ongoing research and debate [7]. Numerous investigations have found that the retention of intraarticular bullet fragments may elevate the probability of heavy metal assimilation and resultant toxicity [8, 9]. Moreover, the persistence of contaminated projectiles or fragments within the joint cavity may precipitate deleterious effects including cartilage impairment, septic arthritis, and accelerated chondrolysis [10]. Instances of intra- or pericapsular bullet retention have been sporadically observed to constrain joint mobility, impede neurovascular structures, and incite synovitis and osteoarthritis [11]. Prolonged retention of bullets has been associated with enduring complications such as infection, chronic pain, and potential migration of the bullet itself [12].

In conclusion, bullet injuries lodged between the ulna and radius represent a unique subset of ballistic trauma with distinct clinical implications. This case highlights the complexity of managing such injuries. The detailed multidisciplinary approach highlighted in this case demonstrates the critical need for collaborative care. The unique trajectory and lodgment of the bullet here also provide valuable insights into the anatomical and functional challenges faced during treatment. This case adds to the literature on ballistic trauma through emphasizing the necessity for tailored surgical strategies and follow-up care. Further research is essential to better understand the prevalence, optimal management, and long-term outcomes of similar injuries, ultimately guiding clinical decision-making and improving patient care in future cases.
